# Rehabilitation of Acute Vs. Chronic Patients With Spinal Cord Injury With a Neurologically Controlled Hybrid Assistive Limb Exoskeleton: Is There a Difference in Outcome?

**DOI:** 10.3389/fnbot.2021.728327

**Published:** 2021-10-27

**Authors:** Amrei Zieriacks, Mirko Aach, Alexis Brinkemper, Daniela Koller, Thomas Armin Schildhauer, Dennis Grasmücke

**Affiliations:** ^1^Department of Spinal Cord Injuries, BG University Hospital Bergmannsheil, Bochum, Germany; ^2^Department of General and Trauma Surgery, BG University Hospital Bergmannsheil, Bochum, Germany; ^3^Institute for Medical Information Processing, Biometrics and Epidemiology, Ludwig-Maximilians-University Munich, Munich, Germany

**Keywords:** spinal cord injury, training, exoskeleton, hybrid assistive limb, rehabilitation

## Abstract

This study aimed to assess the outcome of acute and chronic participants with spinal cord injury (SCI) after 12 weeks of bodyweight supported treadmill training (BWSTT) with a hybrid assistive limb exoskeleton (HAL). Acute participants were defined as ≤12 months between SCI and training, chronic participants >12 months between SCI and training. We assessed whether HAL-assisted BWSTT is advantageous for acute and chronic participants and if length of time post injury impacts the outcome of HAL-assisted BWSTT. As the primary outcome, we assessed the time needed for the 10 meter walk test (10MWT). Hundred and twenty-one individuals participated in a 12-week HAL-assisted BWSTT five times a week. We regularly conducted a 10MWT, a 6 minute walk test (6MWT), and assessed the walking index for spinal cord injury (WISCI II) and lower extremity motor score (LEMS) to evaluate the gait performance without the exoskeleton. Distance and time were recorded by the treadmill while the participant was walking with the exoskeleton. All participants benefit from the 12-week HAL-assisted BWSTT. A significant difference between acute and chronic participants' outcomes was found in 6MWT, LEMS, and WISCI II, though not in 10MWT. Although chronic participants improved significantly lesser than acute participants, they did improve their outcome significantly compared to the beginning. Hybrid assistive limb-assisted BWSTT in the rehabilitation of patients with SCI is advantageous for both acute and chronic patients. We could not define a time related cut-off threshold following SCI for effectiveness of HAL-assisted BWSTT.

## Introduction

The incidence of spinal cord injury (SCI) is 54 per one million people in the USA. There are about 17.81 new cases each year. 66.8% of those are incomplete lesions (47.2% tetraplegic, 19.6% paraplegic) and therefore form the most frequent subgroup since 2015 (National Spinal Cord Injury Statistical Center, 2021). According to the US National Spinal Cord Statistical Center, “less than 1% of patients experienced complete neurological recovery by the time of hospital discharge” (National Spinal Cord Injury Statistical Center, 2021). The quality of life of those patients is strongly dependent on their ability to walk (Brown-Triolo et al., 2002; Estores, 2003; Ditunno et al., 2008). Therefore, functional and motor recovery is of great importance.

Physical activity enhancing motor and neurological recovery of patients with SCI is significantly associated with a higher quality of life, fewer depression symptoms, less SCI-related pain, and better cardiovascular health and fitness (Lam et al., 2014; Warburton et al., 2018; Mehta et al., 2019). Bodyweight supported treadmill training (BWSTT) has been an established method in rehabilitation post-SCI for decades with lasting effects noted for individuals with incomplete injuries (Wirz et al., 2001).

Therapies supporting motor and neurological recovery consist of numerous different approaches such as gait training, BWSTT, robotic-assisted BWSTT, and BWSTT with manual assistance and/ or functional electric stimulation. So far, no approach could prove superiority to the others (Lam et al., 2007, 2014; Schwartz et al., 2011; Mehrholz et al., 2012, 2017; Morawietz and Moffat, 2013).

In the 1980s, the first trials with robotic exoskeletons indicated that robotic-assisted locomotion training in rehabilitation was possible and could improve walking capabilities (van Vliet and Wing, 1991; Colombo et al., 2000). Compared to conventional BWSTT, robotic-assisted locomotion training needs less effort from physiotherapists, enables longer training sessions, and a reproducible gait pattern (Colombo et al., 2000, 2001). Robotic-assisted BWSTT proved several advantages such as improved gait pattern, cardiorespiratory fitness, urinary and bowel functions, and reduced pain and spasticity (Holanda et al., 2017; Brinkemper et al., 2021).

One of the robotic exoskeletons currently utilized in rehabilitation is the hybrid assistive limb (HAL), which uses minimal bioelectrical signals from the remaining motor function of the patients to transfer into movement. To assess patients' with SCI functional gait, the 10 meter walk test (10MWT), 6 minute walk test (6MWT), and walking index for spinal cord injury II (WISCI II) are established and valid measures (van Hedel et al., 2005). Significant improvements as a reduction in the number of steps and increased speed in the 10MWT, walking and cardiovascular endurance in the 6MWT, and lesser walking aids assessed in the WISCI II were reported after HAL exoskeleton training (Kubota et al., 2013; Aach et al., 2014, 2015; Cruciger et al., 2014; Sczesny-Kaiser et al., 2015; Wall et al., 2015; Grasmücke et al., 2017). Improved muscular strength, as measured by the lower extremity motor score (LEMS), has also been reported after HAL-assisted BWSTT (Aach et al., 2014; Sczesny-Kaiser et al., 2015).

Research about robotic assisted BWSTT for patients with SCI is growing, but due to the heterogeneity of different exoskeletons and broad field of applications, the effectiveness is not yet defined. According to a Cochrane review, robotic-locomotor training approaches should be further investigated, specifically which patients and at what stages of recovery the benefit from the intervention is highest (Mehrholz et al., 2012).

The start-time threshold for BWSTT is another topic still to be investigated. The major recovery of patients with acute SCI takes place within the first 12 months of rehabilitation (Fawcett et al., 2007). Still, significant changes occur in chronic patients with SCI (trauma >12 months ago) with a small degree of neurologic recovery (Piepmeier and Jenkins, 1988; Kirshblum et al., 2004). According to Lam et al., bodyweight-supported training seems to be more effective in acute patients than in chronic patients (Lam et al., 2007). According to Grasmücke et al., patients with chronic SCI benefit from HAL-assisted BWSTT (Grasmücke et al., 2017). Patients with incomplete SCI have greater chances of neurological and functional recovery compared to patients with complete SCI (Fawcett et al., 2007; Curt et al., 2008; Lee et al., 2016).

The purpose of our study was to investigate the impact of HAL-assisted BWSTT on functional and motor recovery in post-acute phases of neurorehabilitation. We hypothesize that there will be significant improvements in gait and motor function after HAL BWSTT as measured using the 10MWT, 6MWT, WISCI II, and LEMS outcome measures for participants with both acute and chronic SCI. We also hypothesize that patients with acute injury will improve motor function to the same extent as patients with chronic injury.

## Materials and Methods

### Patient Population

Our cohort consists of participants that started HAL training between 02/2012 and 10/2018 at Bergmannsheil Hospital in Bochum, Germany.

Both acute and chronic participants with SCI were included, with time between trauma and therapy of 12 months marking the cut-off between acute and chronic using previous guidelines by Fawcett et al. (2007).

The data of chronic and acute participants have been described previously in our subgroup analysis and a paper currently under review (Grasmücke et al., 2017).

Inclusion criteria were defined as incomplete SCI (AIS C, D) or complete SCI from conus medullaris to cauda equine with zones of partial preservation (ZPP) (AIS A) and existing motor function of hip and knee extensor and flexor muscle groups to operate the exoskeleton (Frankel and Janda Grade 1/5 or 2/5) (Janda, 1983).

Exclusion criteria consisted of: inadequate attendance of training sessions such that participants were only present for ≤ 20, a severe joint contracture in hip or knee, bodyweight over 100 kg, pressure sores, non-consolidated fractures, epilepsy, cognitive impairment that makes the therapy substantially difficult or impossible, and severe heart insufficiency. All 121 participants were classified according to the International standards for neurological classification of spinal cord injury (ISNCSCI) by the American spinal cord injury association (ASIA) impairment scale (AIS) (Kirshblum et al., 2011).

The participants suffered spinal cord lesions between C2 and L4 (32 cervical, 55 thoracic, 34 lumbal). The mean time between SCI and the start of HAL training was 65.3 months (SD 89.5 months, range: 0–396 months).

All participants gave written informed consent to participate in this trial. They confirmed consent for anonymized data publishing. The ethics committee of BG University Hospital Bergmannsheil and the Ruhr University Bochum approved the study protocol. This study was conducted according to the principles expressed in the Declaration of Helsinki.

### The Exoskeleton

The HAL (Cyberdyne Inc., Japan) exoskeleton used in this study acts on the lower limbs. Electrical motors support flexion and extension of the hip and knee joints. The robot suit is attached to the patient's legs and around the waistline. EMG electrodes detect voluntary minimal bioelectrical signals from extensor and flexor muscles of the hip and knee. Thereby, the gait pattern is controlled by the patient itself and allows an individual motion sequence (Suzuki et al., 2007). Hybrid assistive limb locomotion training is safe for patients with SCI and enhances the rehabilitation progress (Kubota et al., 2013; Aach et al., 2014, 2015; Cruciger et al., 2014; Sczesny-Kaiser et al., 2015; Wall et al., 2015; Grasmücke et al., 2017).

### Intervention

All participants used the HAL robot suit exoskeleton under the supervision of a HAL-trained physiotherapist. The training took place five times a week for 90–120 min for 3 months. In addition to the HAL-based training, the participants regularly performed a 10MWT and 6MWT without the exoskeleton with individual walking aids. The treadmill-associated data, distance, and time were continuously recorded. The exoskeleton's weight (14–17 kg depending on the size) and an individual amount of the patient's body weight (0–20 kg) were compensated by the bodyweight support of the treadmill. The treadmill used is by Woodway, Inc.

### Outcome Measures

The physiotherapists performing the examinations and training supervision did not participate in data analysis or study design.

The functional outcome and walking capability of the participants were measured at the beginning, after 6 and 12 weeks of HAL training. For the 10MWT, the seconds needed for walking a 10 m distance were recorded (van Hedel et al., 2005, 2006). The participants were advised to walk the 10 m at their own preferred pace. The required assistance in the 10MWT was monitored by the WISCI II (Ditunno et al., 2000; Ditunno and Dittuno, 2001). Through 6MWT, walking and cardiovascular endurance was assessed by the distance participants walked in 6 min. Participants chose their speed and were advised to pause if they felt unable to continue (Harada et al., 1999; van Hedel et al., 2006). The LEMS was recorded before and after the 12 weeks of training to evaluate the motor function and gait performance (Waters et al., 1994).

The parameters of walking time and distance were recorded by the treadmill during each training session while wearing the exoskeleton. The velocity of the treadmill was settled individually among comfortable and maximum speed tolerated by the participants.

### Statistical Analysis

Descriptive statistics of age, injury characteristics, and sex were calculated by frequency distributions for categorical data and means for continuous variables.

All outcomes were tested for normal distribution using the Shapiro-Wilk Test and visibly through distribution plots for all three time points. Time trends over all measurements were visualized for all the outcomes WISCI II, 10MWT, 6MWT, and LEMS. To analyze the differences between chronic and acute participants and the differences over the three time points of evaluation (start of training, after 6 weeks, and after 12 weeks), a repeated measures ANOVA was performed for each outcome (testing two groups over three time-points). For the repeated measures ANOVA, cases were only included if they had valid measures for all three timepoints. Equal group variances were tested with the Levene's test. The alpha level was set to 0.05.

Differences within and between groups have been estimated with *t*-tests and with repeated measure ANOVA, assuming normal distribution of the continuous variable. Data was lightly skewed at some timepoints; therefore tests have been repeated with a Wilcoxon test (for non-parametric data). Results from the Wilcoxon test have confirmed the results of the *t*-test. Levene's tests for the repeated measures ANOVA were all non-significant.

All statistical analyses have been performed with SAS for Windows (Vers. 9.4).

## Results

A total of 137 patients were screened for the study. Ten patients were excluded after the first screening, and six participants were eliminated for evaluation because they had participated in <20 sessions. The 121 participants included in the analyses are presented in Table 1.

**Table 1:** Participants characteristics.

**Category**		
Sex	Male	89 participants
	Female	31 participants
Age	Mean	44.3 years
	Range	16–74 years
Time since SCI	Mean	65.3 months
	Range	0–396 months
Subgroups	Acute	47 participants
	Chronic	74 participants
Lesion level	Cervical	32 participants
	Thoracic	55 participants
	Lumbal	34 participants
AIS	AIS A with ZPP	24 participants
	AIS C	61 participants
	AIS D	36 participants

The participants were divided into two subgroups (acute, chronic) according to the time between the date of SCI and the point of time training started. The acute group consisted of participants with a SCI duration under 12 months at the beginning of the intervention (*n* = 47). In the chronic group were 74 participants with the SCI being more than 1 year ago (13 months to 33 years).

Within the time of training (02/12 to 10/18), no adverse events happened. There were no falls or discontinuation due to HAL-assisted BWSTT. All Means and standard deviations are displayed in Table 2.

**Table 2:** Means, standard deviations, and significance.

**Test**	**Group**	**Mean start**	**Standard deviation start**	**Mean end**	**Standard deviation end**	**Significance within group**	**Significance between groups**
Time walking on the treadmill with the exoskeleton	Acute	15.9 min	5.8 min	28.9 min	6.5 min	*p* ≤ 0.0001	
	Chronic	14.6 min	6.3 min	30.0 min	4.3 min	*p* ≤ 0.0001	
	All	15.1 min	6.1 min	29.6 min	5.3 min	*p* ≤ 0.0001	*p* = 0.1642
Distance covered on the treadmill with the exoskeleon	Acute	261.7 m	183.8 m	996.6 m	429,7 m	*p* ≤ 0.0001	
	Chronic	220.5 m	165.1 m	926.6 m	403.3 m	*p* ≤ 0.0001	
	All	236.3 m	172.9 m	953.5 m	413.2 m	*p* ≤ 0.0001	*p* = 0.1987
10MWT	Acute	63.6 s	47.2 s	26.3 s	37.5 s	*p* ≤ 0.0001	
	Chronic	68.7 s	59.3 s	34.5 s	30.2 s	*p* ≤ 0.0001	
	All	70.1 s	66.0 s	36.5 s	43.4 s	*p* ≤ 0.0001	*p* = 0.7161
6MWT	Acute	127.1 m	93.6 m	245.1 m	133.7 m	*p* ≤ 0.0001	
	Chronic	111.5 m	102.4 m	158.8 m	116.7 m	*p* ≤ 0.0001	
	All	115.5 m	98.3 m	185.2 m	129.5 m	*p* ≤ 0.0001	*p* ≤ 0.0001
WISCI II	Acute	7.2	4.8	13.6	5.4	*p* ≤ 0.0001	
	Chronic	9.3	5.5	11.3	4.6	*p* ≤ 0.0001	
	All	8.5	5.3	12.2	5.0	*p* ≤ 0.0001	*p* ≤ 0.0001
LEMS	Acute	28.4	9.8	37.1	9.8	*p* ≤ 0.0001	
	Chronic	22.3	9.6	25.4	10.7	*p* ≤ 0.0001	
	All	24.7	10.1	30.2	11.8	*p* ≤ 0.0001	*p* ≤ 0.0001

### HAL Associated Outcomes

All participants improved treadmill performance within the 12 weeks of training.

Participants could significantly extend walking time with the exoskeleton on the treadmill from 15.1 min (±6.1 min) baseline to 29.6 min (±5.3 min) after 12 weeks (*p* ≤ 0.0001). Acute participants walked from 15.9 min (±5.8 min) baseline to 28.9 min (±6.5 min) after 12 weeks (*p* ≤ 0.0001). Chronic participants walked from 14.6 min (±6.3 min) baseline to 30.0 min (±4.3 min) after 12 weeks (*p* ≤ 0.0001). Due to the limited session time, participants were not allowed to walk longer than 30 min. No significant difference in walking time could be observed between the subgroups (*p* = 0.1642).

Ambulated distance increased significantly in all participants from 236.3 m (±172.9 m) baseline to 953.5 m (±413.2 m) after 12 weeks (*p* ≤ 0.0001). Acute participants improved from 261.7 m (±183.8 m) baseline to 996.6 m (±429,7 m) after 12 weeks (*p* ≤ 0.0001). Chronic participants improved from 220.5 m (±165.1 m) baseline to 926.6 m (±403.3 m) after 12 weeks (*p* ≤ 0.0001). Concerning walking distance, no significant difference was measured between the subgroups (*p* = 0.1987).

### Functional Outcomes

All participants significantly improved in the functional assessments performed without the exoskeleton. Table 3 demonstrates the included and excluded participants for all functional tests due to AIS levels.

**Table 3:** Functional tests divided according to AIS.

	**All**	**AIS A**	**AIS C**	**AIS D**
Participants included in 10MWT	111	21	56	34
Participants excluded in 10MWT	10	3	5	2
Sum	121	24	61	36
Participants included in 6MWT	88	17	47	24
Participants excluded in 6MWT	33	7	14	12
Participants included in WISCI II	116	22	59	35
Participants excluded in WISCI II	5	2	2	1
Participants included in LEMS	109	23	55	31
Participants excluded in LEMS	12	1	6	5

#### 10MWT

**Figure 1:**
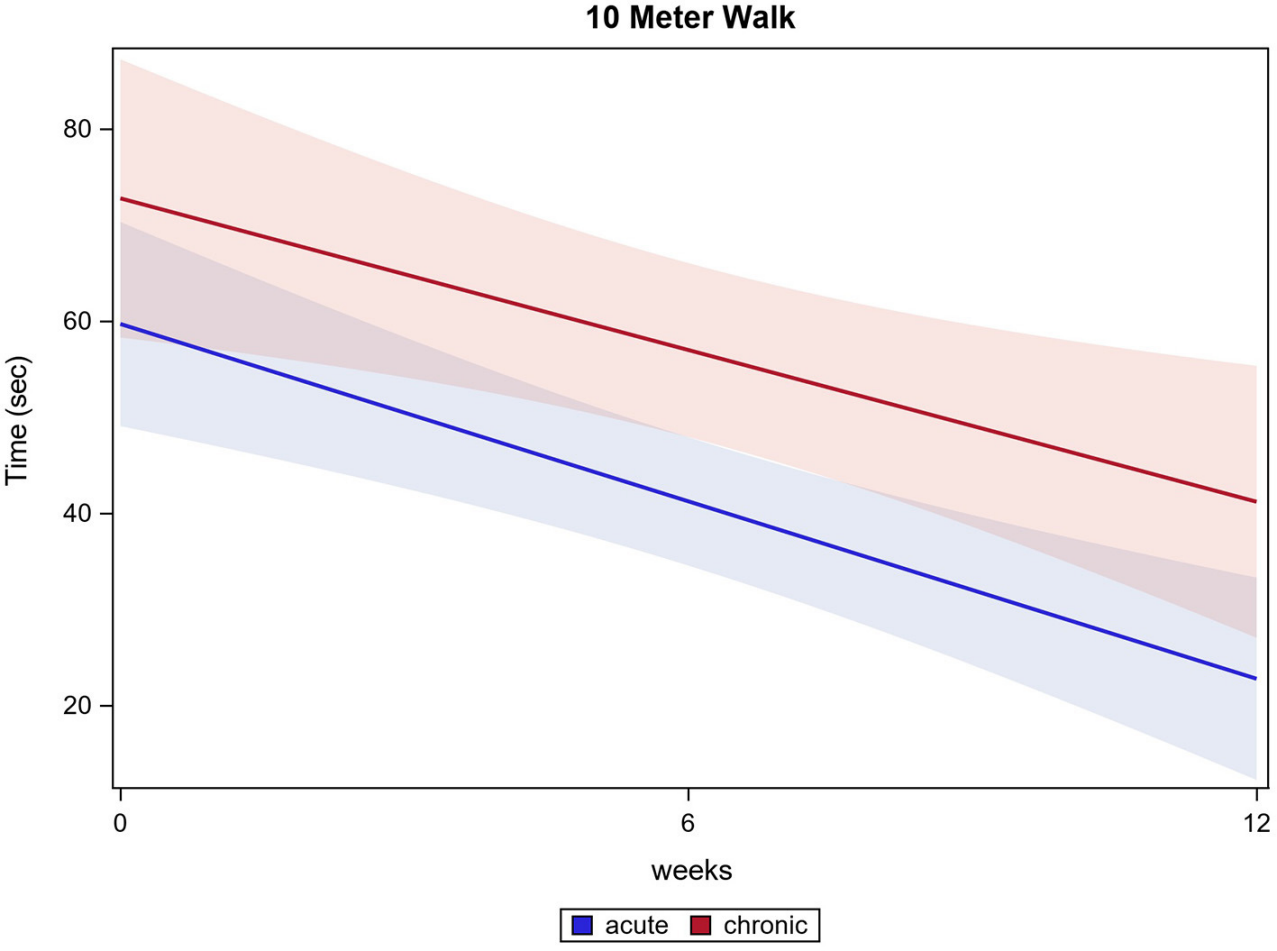
Ten meter walk.

Of all 121 participants, 111 participants were able to conduct the 10MWT at the first training session, 117 participants completed the 10MWT after 12 weeks of training. Participants significantly accelerated in 10MWT from 70.1 s (±66.0 s) baseline to 36.5 s (±43.4 s) after 12 weeks (*p* ≤ 0.0001). Acute participants improved from 63.6 s (±47.2 s) baseline to 26.3 s (±37.5 s) after 12 weeks and chronic participants improved from 68.7 s (±59.3 s) baseline to 34.5 s (±30.2 s) after 12 weeks (both *p* ≤ 0.0001). There were no significant differences between the two groups in the 10MWT (*p* = 0.7161). 10MWT results are shown in Figure 1.

#### 6MWT

**Figure 2:**
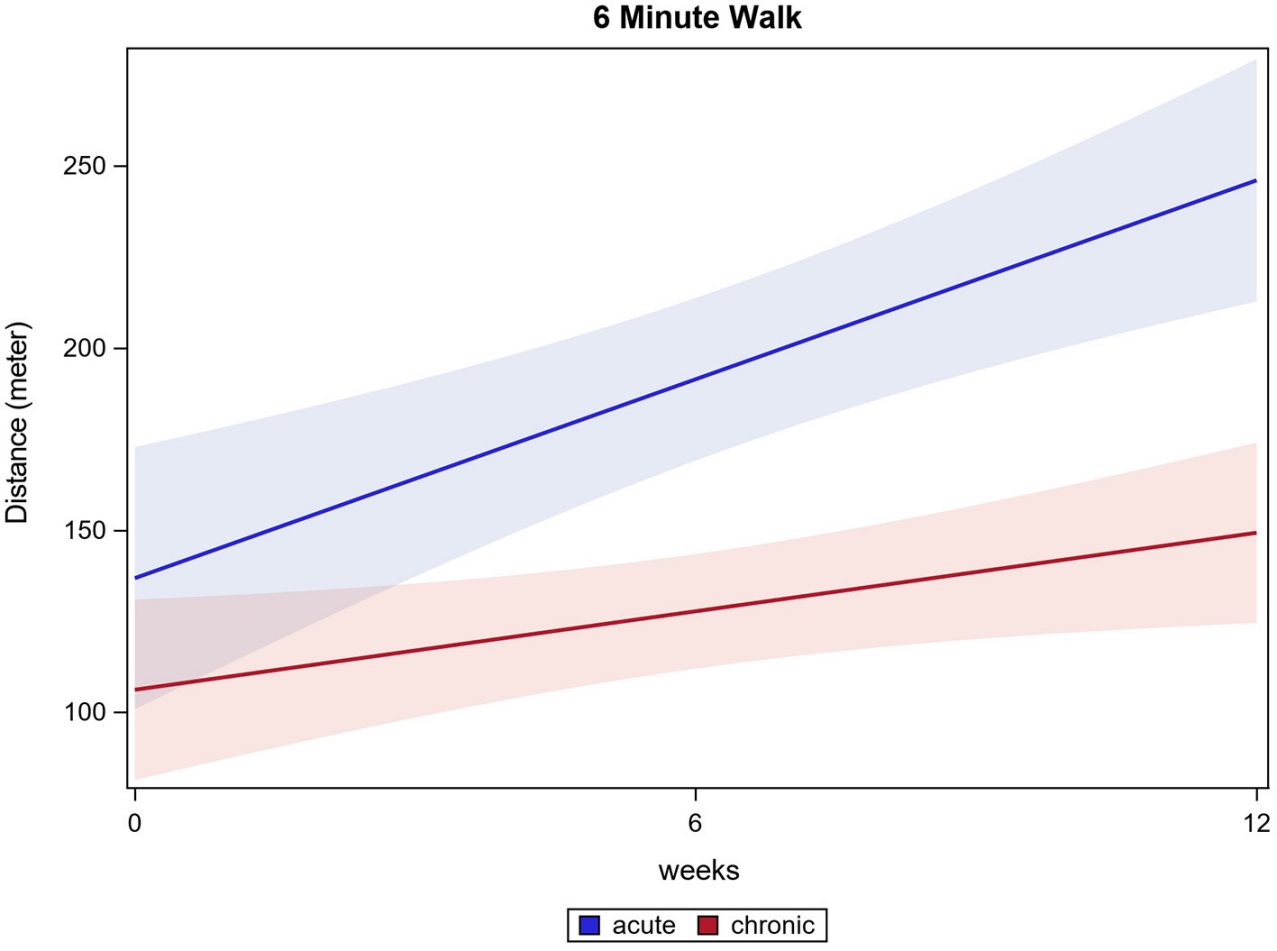
Six minute walk.

Eighty-eight participants had fully documented 6MWT values for all time points and were included in the analysis. The distance ambulated in 6 min increased significantly in all participants from 115.5 m (±98.3 m) baseline to 185.2 m (±129.5 m) after 12 weeks (*p* ≤ 0.0001). Acute participants improved from 127.1 m (±93.6 m) baseline to 245.1 m (±133.7 m) after 12 weeks. Chronic participants improved from 111.5 m (±102.4 m) baseline to 158.8 m (±116.7 m) after 12 weeks (both *p* ≤ 0.0001). Acute participants improved significantly more than chronic participants, while both groups enhanced the performance in 6MWT (*p* ≤ 0.0001). 6MWT results are shown in Figure 2.

#### WISCI II

**Figure 3:**
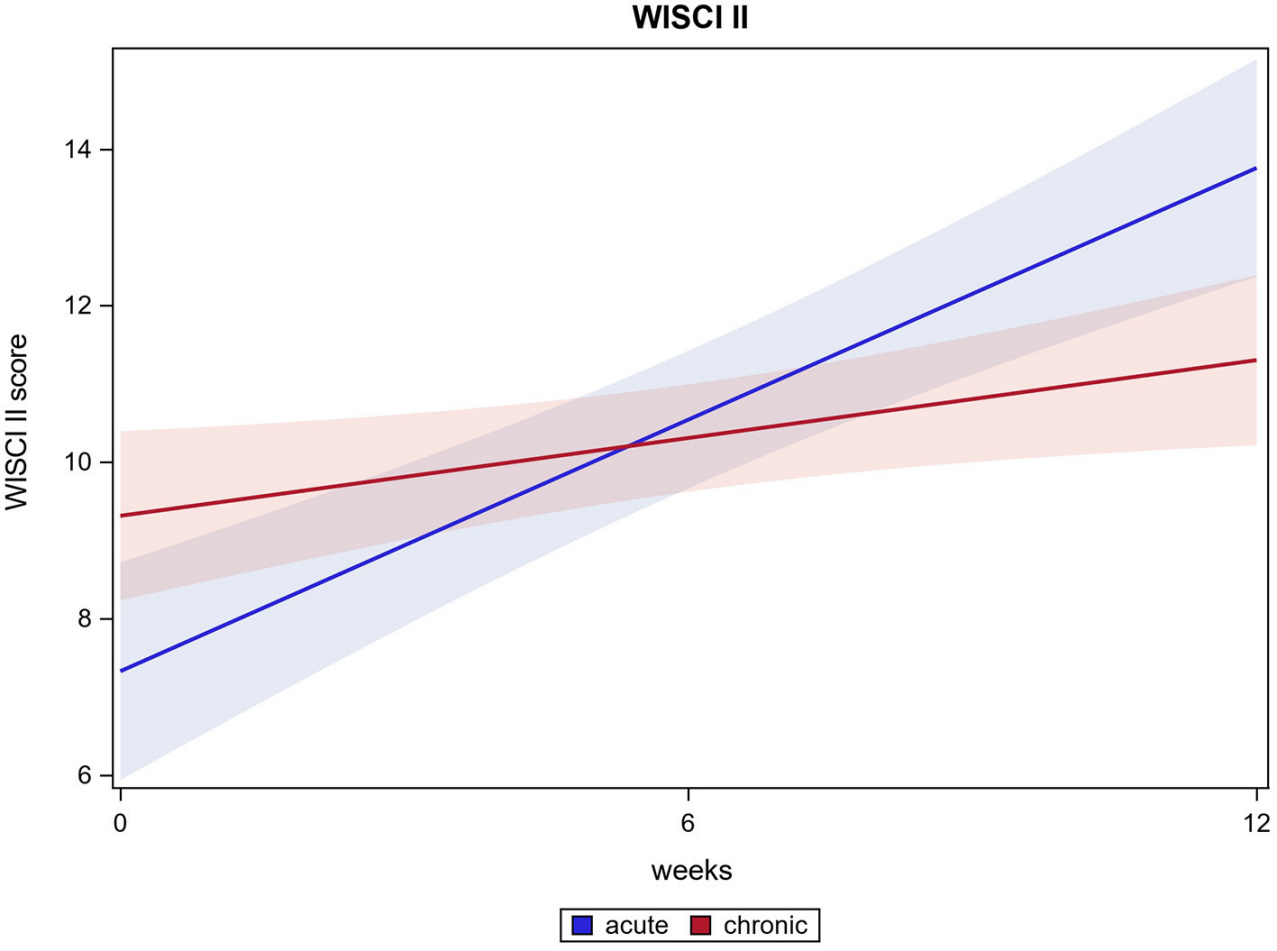
WISCI II score.

For 116 out of 121 participants the WISCI II could be measured after HAL training. The Mean score from 8.5 (±5.3) at baseline improved to 12.2 (±5.0) after 12 weeks (*p* ≤ 0.0001). Acute participants' WISCI II scores changed from 7.2 (±4.8) at baseline to 13.6 (±5.4) after 12 weeks, chronic participants' WISCI II scores from 9.3 (±5.5) at baseline to 11.3 (±4.6) after 12 weeks (both *p* ≤ 0.0001). There was a significant difference between acute and chronic participants' results. Whereas, both groups improved, acute participants' improvement is significantly higher than chronic patient's improvement. (*p* ≤ 0.0001). WISCI II score results are shown in Figure 3.

#### LEMS

**Figure 4:**
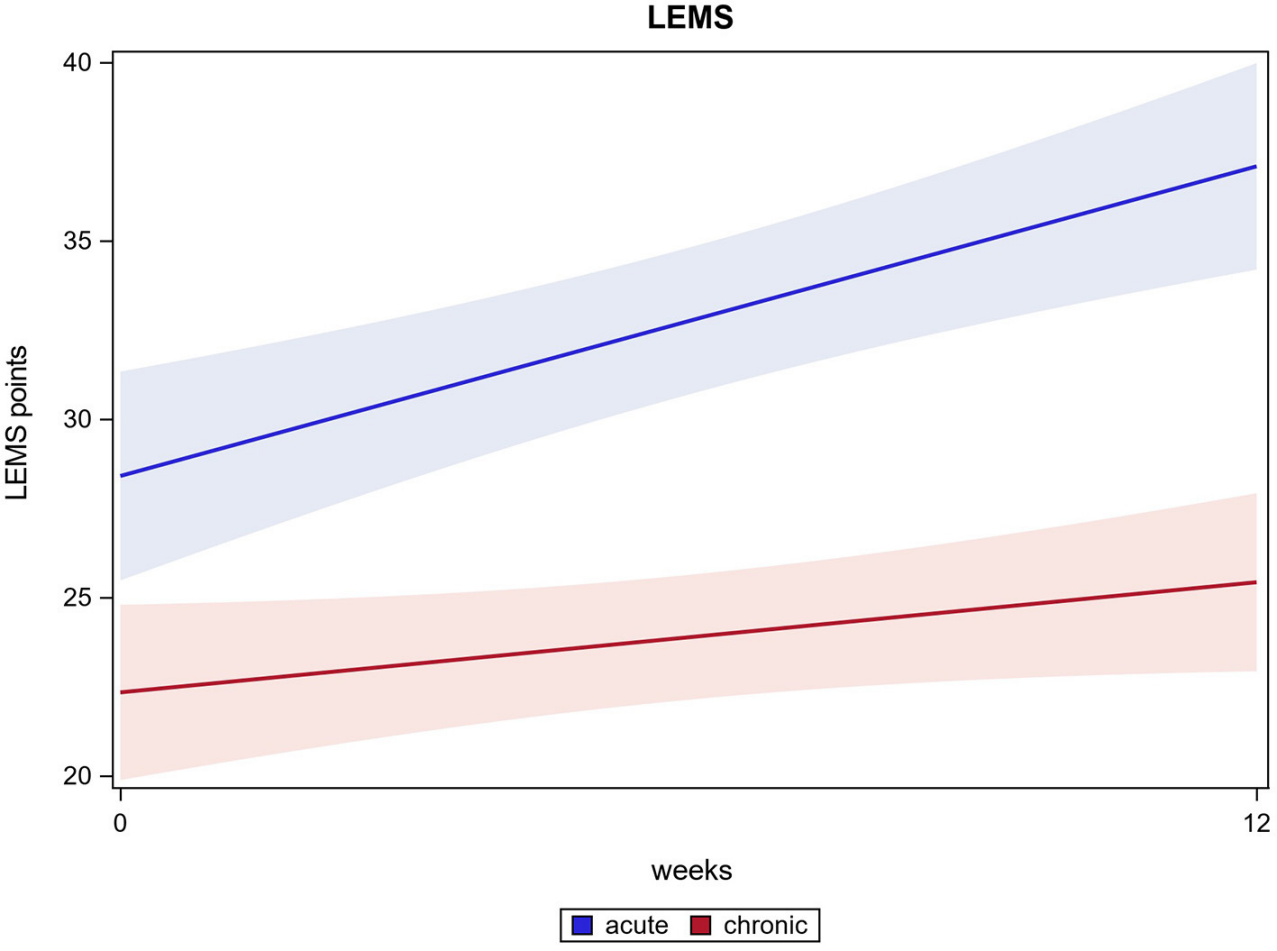
LEMS.

In 109 out of 121 participants, an enhanced LEMS could be examined at two time points (at baseline and after 12 weeks). The scores improved significantly from 24.7 (±10.1) at baseline to 30.2 (±11.8) after 12 weeks (*p* ≤ 0.0001). Acute participants improved significantly from 28.4 (±9.8) at baseline to 37.2 (±9.8) after 12 weeks, chronic participants improved significantly from 22.3 (±9.6) at baseline to 25.4 (±10.7) after 12 weeks (both *p* ≤ 0.0001). There was a significant difference found between acute and chronic participants' improvement, while both groups improved significantly from the start of the intervention to the end (*p* ≤ 0.0001). LEMS results are shown in Figure 4.

## Discussion

This study investigated whether HAL-assisted BWSTT is advantageous for acute and chronic patients with SCI and if there was a cut-off threshold for effectiveness.

We investigated 121 acute vs. chronic participants performing a 12-week HAL-supported BWSTT.

The present study demonstrated that acute and chronic participants benefit in all conducted tests after 12 weeks of HAL-assisted BWSTT. For 6MWT, WISCI II, and LEMS, test results show a significant difference between the acute and the chronic participants' outcomes. For the 6MWT, acute participants improved distance walked by 93% and chronic participants improved in means by 42% (equates 47 m). Bohannon et al. state that a clinically relevant change in 6MWT is considered as at least 14 to 30.5 m gain in 6MWT (Bohannon and Crouch, 2017). Following this review, acute and chronic participants outcomes could be considered clinically relevant, although acute participants improved to a greater extend. Most of our participants are wheelchair users. The preserved motor function enables them to ambulate short distances, they would, however, use a wheelchair for long distances to save time. Therefore, the 6MWT is measured as an objective means to assess a patient's walking, muscular and cardiovascular endurance although the everyday relevance is limited. Ten meters are a distance likelier to be ambulated by foot. Therefore, the 10MWT presents a more clinically relevant outcome concerning speed of ambulation. The 6MWT is described to be potentially biased as not all participating individuals could perform the test. Therefore, a smaller number of participants are considered. In our data, 33 participants did not perform the 6MWT at a minimum of one time point. Generally, it must be stated that acute participants started training with overall better mean scores at most tests. Moreover, one should assess the outcome of acute participants considering spontaneous recovery. Still, improvement was visible in both chronic and acute participants for all functional tests.

Both, acute and chronic participants significantly improved their test values in 10MWT. Surprisingly, there was no significant difference between acute participants' and chronic participants' outcomes in 10MWT and all treadmill-associated data.

Commencing HAL-associated BWSTT seems to be of advantage in the acute phase after SCI and after several years. As described in our previous paper, chronic patients benefit from HAL-associated BWSTT with improved overground walking and mobility, and no adverse events were recorded (Grasmücke et al., 2017). Whilst feasibility was proved for chronic participants, there is to the best of our knowledge no study comparing acute and chronic participant's outcomes after HAL-assisted training.

Concerning the limitations of this study, we did not record additional treatments or medication for our participants. Most acute participants attended physiotherapy five times a week, some including gait training and some without. Chronic participants attended between zero and three times a week of physiotherapy. Some participants attended regular wheelchair sports groups. The spectrum of different additional treatments is so wide, that standardization and comparison were not possible. As the potential for rehabilitation is highest in the first year after SCI, it would be ethically unjustifiable to prohibit additional treatments to acute participants in particular. Participants were asked to continue any established treatments and alter medication only in case of need.

Another limitation is, that a ceiling effect has been described for higher AIS graded patients (C, D). That may cause inaccuracies in measuring the effect of the treatment. It can be balanced by the functional tests WISCI II, 10MWT, and 6MWT we conducted (Steeves et al., 2007).

The lack of a control group is a limitation of this study. A randomized controlled trial to further assess and compare the improvements in motor function with HAL training is planned for future studies. This should help to eliminate potential biases like placebo effect or investigator bias (Lammertse et al., 2007). Hybrid assistive limb training should be compared to conventional training and gait machines with other means of control, such as the Lokomat (Wall et al., 2015).

## Conclusion

This study provides evidence that a training protocol with BWSTT with assisted HAL training improves functional ambulation in all participants. It indicates, that acute as well as chronic participants with SCI enhance their functional ambulation without the exoskeleton. While further investigation is needed to compare such training to a control group using randomization, these findings are a first step toward discovering a walking protocol for individuals with SCI regardless of chronicity.

## Data Availability Statement

The original contributions presented in the study are included in the article/supplementary material, further inquiries can be directed to the corresponding author/s.

## Ethics Statement

The studies involving human participants were reviewed and approved by the Ethics Committee of BG University Hospital Bergmannsheil and the Ruhr University Bochum. Written informed consent to participate in this study was provided by the participants' legal guardian/next of kin.

## Author Contributions

AZ: conceptualization, methodology, investigation, writing—original draft, visualization, and project administration. MA: conceptualization, methodology, investigation, reviewing and editing, visualization, and project administration. AB: conceptualization, methodology, and writing—reviewing and editing. DK: formal analysis, data curation, writing—reviewing and editing, and visualization. TS: conceptualization, writing—reviewing and editing, and supervision. DG: conceptualization, methodology, writing—reviewing and editing, and supervision. All authors have read and approved the final manuscript.

## Funding

This work was supported by the New Energy and Industrial Technology Development Organization, Japan (NEDO) and a governmental grant (I&K-Gender-Study, European-Union, and NRW, Germany under Grant number 005-GW02-069A). The trial has been exclusively performed and supervised by the staff of BG University Hospital Bergmannsheil Bochum.

## Conflict of Interest

The authors declare that the research was conducted in the absence of any commercial or financial relationships that could be construed as a potential conflict of interest.

## Publisher's Note

All claims expressed in this article are solely those of the authors and do not necessarily represent those of their affiliated organizations, or those of the publisher, the editors and the reviewers. Any product that may be evaluated in this article, or claim that may be made by its manufacturer, is not guaranteed or endorsed by the publisher.

## Abbreviations

SCI: spinal cord injury
HAL: hybrid assistive limb exoskeleton
BWSTT: bodyweight supported treadmill training
10MWT: 10 meter walk test
6MWT: 6 minute walk test
LEMS: lower extremity motor score
WISCI II: walking index for spinal cord injury II
ASIA/AIS: American spinal injury association/Asia impairment scale
ZPP: zones of partial preservation.
